# Rosmarinic Acid Alleviates the Endothelial Dysfunction Induced by Hydrogen Peroxide in Rat Aortic Rings via Activation of AMPK

**DOI:** 10.1155/2017/7091904

**Published:** 2017-08-13

**Authors:** Hui Zhou, Baocai Fu, Bo Xu, Xiangquan Mi, Gang Li, Chengjun Ma, Jianxin Xie, Ji Li, Zhenhua Wang

**Affiliations:** ^1^Center for Mitochondria and Healthy Aging, College of Life Sciences, Yantai University, Yantai 264005, China; ^2^Intensive Care Unit, Yantaishan Hospital, Yantai 264001, China; ^3^School of Medicine, Shihezi University, Shihezi 832002, China

## Abstract

Endothelial dysfunction is the key player in the development and progression of vascular events. Oxidative stress is involved in endothelial injury. Rosmarinic acid (RA) is a natural polyphenol with antioxidative, antiapoptotic, and anti-inflammatory properties. The present study investigates the protective effect of RA on endothelial dysfunction induced by hydrogen peroxide (H_2_O_2_). Compared with endothelium-denuded aortic rings, the endothelium significantly alleviated the decrease of vasoconstrictive reactivity to PE and KCl induced by H_2_O_2_. H_2_O_2_ pretreatment significantly injured the vasodilative reactivity to ACh in endothelium-intact aortic rings in a concentration-dependent manner. RA individual pretreatment had no obvious effect on the vasoconstrictive reaction to PE and KCl, while its cotreatment obviously mitigated the endothelium-dependent relaxation impairments and the oxidative stress induced by H_2_O_2_. The RA cotreatment reversed the downregulation of AMPK and eNOS phosphorylation induced by H_2_O_2_ in HAEC cells. The pretreatment with the inhibitors of AMPK (compound C) and eNOS (L-NAME) wiped off RA's beneficial effects. All these results demonstrated that RA attenuated the endothelial dysfunction induced by oxidative stress by activating the AMPK/eNOS pathway.

## 1. Introduction

The vascular endothelium plays critical roles in maintaining the vascular structure and function [1]. In physiological states, the endothelium releases both relaxing and contracting factors including nitric oxide (NO), prostacyclin, and endothelin, which contribute to the local regulation of vascular tone and the coagulation [2].

Endothelial cells also secrete the reactive oxygen species (ROS), especially the hydrogen peroxide (H_2_O_2_), as the fast diffusion signal to recruit the leukocytes to the injury site and the endothelial NADPH oxidase is the main source of ROS [3]. Whereas excess ROS will result in oxidative stress which contributes to vascular dysfunction in cardiovascular events [4], diabetes [5], stroke [6], atherosclerosis [7], and so forth, it is becoming increasingly clear that oxidative stress contributed to the development of the macrovascular complications [8]. Indeed, recent studies have shown that the mechanism of endothelial dysfunction is largely due to the reduced bioavailability of endothelium-derived NO by oxidative stress [9]. The presence of ROS not only reduces the bioavailability of NO [10] but also results in the eNOS uncoupling which will result in more ROS formation [11].

Accumulating evidences from bench to bed support the free radical scavenging properties of phenolic antioxidants and the pharmacological activities against oxidative stress-mediated vascular disorders. Such (cases?) as resveratrol [12], curcumin [13], and the flavanol (−)-epicatechin [14] showed widely protective effects on the endothelial cells *in vivo* and *in vitro*. Rosmarinic acid is one of the most potent antioxidants among the simple phenolic compounds [15]. Rosmarinic acid (*α*-O-caffeoyl-3, 4-dihydroxyphenyl lactic acid; RA) is a natural phenol antioxidant contained in some Labiatae family plants used in traditional medicine and phytotherapy such as *Perilla frutescens* (L.) Britt., *Salvia miltiorrhiza* Bge., *Rosmarinus officinalis* L., and *Lavandula angustifolia* Mill. RA possesses many bioactivities including antioxidative, astringent, anti-inflammatory, antimicrobial, antiangiogenic, antiviral, antirheumatic, antiallergic, antidepressant, antidiabetic, and antitumor effects [16]. Sotnikova et al. proved that RA improved the reactivity to phenylephrine of aortic rings and prevented the upregulation of IL-1*β*, TNF-*α*, and endothelin pathway in diabetic rats *in vivo* [8], while the effects on endothelium-dependent vasodilation were not investigated. Here, we established an oxidative injury with hydrogen peroxide (H_2_O_2_) and investigated the protective effects of RA on endothelium-dependent vasodilation mediated by acetylcholine (ACh) and the underlying mechanisms. Our results demonstrated that the protective activities of RA are mediated by an AMPK-eNOS signaling pathway.

## 2. Materials and Methods

### 2.1. Chemicals

Rosmarinic acid, acetylcholine (ACh), compound C,5-aminoimidazole-4-carboxamide-1-*β*-D-ribofuranoside (AICAR), apocynin, and diphenyliodonium were purchased from Sigma Chemical Co. (St. Louis, MO, USA); phenylephrine (PE) and *l*-N-nitro arginine methyl ester (L-NAME) were purchased from Aladdin Industrial Co. (Shanghai, China). The other reagents were of analytical purity.

### 2.2. Animals

Three-month-aged male Wistar rats (200–250 g) were obtained from the Animal Center of Shandong Luye Pharmaceutical Co. Ltd. (Yantai, China). The rats were maintained in a 12 h light/dark cycle and had free access to food and water. All experimental procedures were approved by the Institutional Animal Care and Use Committee of National Institute of Pharmaceutical Education and Research.

### 2.3. Preparation of Rat Aortic Rings

The thoracic aorta was isolated and placed in 4°C modified Krebs-Henseleit (K-H) solution (mM: NaCl, 118; KCl, 4.7; KH_2_PO_4_, 1.2; MgSO_4_, 1.2; NaHCO_3_, 25.0; CaCl_2_, 2.5; D-glucose, 10.0. pH 7.4, [17]). The excess connective tissue was carefully cleaned and the aorta was cut into segments approximately 3 mm long. In some experiments, the aortic endothelium was removed by the paper clip. The tension of the aortic ring was recorded with a linear force transducer, and the K-H solution was aired with a 95% O_2_ and 5% CO_2_ mixture and maintained at 37°C. All the vessels were equilibrated for 1 h and the basic tension was adjusted to 2.0 g before the experiment. During the equilibration period, the K-H solution was replaced every 15 min. At the beginning of an experiment, the aortic rings were exposed to 80 mM KCl for 3 times until the responses were stable. The intact endothelium function was verified by the relaxation reaching more than 85% induced by ACh (10 *μ*M) to induce in the precontracted aorta rings with PE (1 *μ*M). The endothelium was considered effectively removed when the relaxation was less than 10% induced by ACh.

### 2.4. Endothelial Dysfunction Induced by H_2_O_2_ and the RA Treatment in Rat Aortic Rings

After 10 min equilibration with the new K-H solution, the aortic rings were pretreated with various concentrations of H_2_O_2_ (2.5, 5.0, and 10.0 mM) for 10 min. Following washout of H_2_O_2_, the aortic rings were depolarized with 80 mM KCl for 2 times. After returning to baseline tension, the rings were allowed to equilibrate for 20 min, and then the contraction were induced with PE (1 *μ*M) till a stable plateau in tension. Then, each ring was exposed to increasing concentration of ACh (10^−3^, 10^−2^, 10^−1^, 1, 5, 10, 50 *μ*M) to generate a dose-dependent relaxation response. In the RA intervention experiment, the aortic rings were incubated with various concentrations (50.0 *μ*M, 25 *μ*M, 12.5 *μ*M) of RA 10 min prior to exposure to 5 mM H_2_O_2_. Thereafter, a second vasodilation reactivity to ACh was obtained to evaluate the integrity of the endothelium after PE-induced contraction. In order to investigate the roles of AMPK in H_2_O_2_-induced endothelium dysfunction, the aortic rings were separately pretreated for 10 min with AMPK inhibitor (compound C) and AMPK activator (AICAR) before the exposure to H_2_O_2_ (5 mM).

### 2.5. Measurement of H_2_O_2_ Caused the Vasocontraction Impairment Mediated by Smooth Muscle Cells

In order to exclude the vasocontraction impairment mediated by smooth muscle cells injury, the vasoconstriction reactivity to PE was investigated after 5 mM H_2_O_2_ treatment in endothelial-intact (EC+) or endothelial-denuded (EC−) aortic rings.

### 2.6. Detection of O_2_^−^ by NBT Reduction Assay

NBT reduction assay was performed as the method described previously [18]. Briefly, the aortic rings were incubated with the K-H solution containing 100.0 *μ*M NBT for 1 h after the experiment. Subsequently, the HCl (0.5 mM) was added to stop the reaction. Then, the aortic rings were washed 3 times with PBS buffer; after they were minced and centrifuged at 20000*g* for 20 min on the part of the mixture of 40 mg/L diethylenetriaminepentaacetic acid, which was dissolved into 0.1 M NaOH and 0.1% SDS, the pellet was suspended in 0.5 mL of pyridine, along with being heated at 80°C for 1.5 h in order to extract formazan. The mixture was experienced a second centrifugation at 10000*g* for 10 min. Optical density (OD) was measured at 540 nm.

### 2.7. Cell Culture and Treatment

HAEC (human aortic endothelial cells) were purchased from Cell Bank, Shanghai Institutes for Biological Sciences, Chinese Academy of Sciences, Shanghai, and cultured in Dulbecco's Modified Eagle Medium (GIBCO) supplemented with L-glutamine, pyridoxine hydrochloride, 110 mg/L sodium pyruvate, 100 U/mL penicillin, and 100 *μ*g/mL streptomycin and amphotericin at 37°C in a humidified atmosphere of 5% CO_2_. Cells cultured up to six or fewer passages were first grown to confluence before exposure to H_2_O_2_ (5 mM) for 10 min, and stimulated by RA (50 *μ*M) containing H_2_O_2_ (5 mM) for 10 min, to clarify the activity of AMPK on the expression of the phosphor-eNOS. Therefore, cells were treated with compound C (inhibitor of AMPK) with H_2_O_2_ and in the presence of RA for 10 min.

### 2.8. Western Blotting Assay

After lysis of the cells, the protein samples (25 *μ*g/lane) were resolved by electrophoresis on 10% sodium dodecyl sulfate (SDS) polyacrylamide gels and then transferred to nitrocellulose membranes. The membranes were incubated in blocking buffers and then incubated with more of the following primary antibodies: anti-AMPK (1 : 1000, Cell Signaling Technology, MA, USA), anti-phospho-AMPK (Thr172) (1 : 1000, Cell Signaling Technology), anti-phospho endothelial nitric oxide synthase (eNOS, Ser1177), and anti-endothelial nitric oxide synthase (1 : 1000, Cell Signaling Technology). Thereafter, the membranes were washed and incubated with horseradish peroxidase-conjugated secondary antibodies. (1 : 2000, Cell Signaling Technology).

### 2.9. Statistical Analysis

Results were expressed as the mean ± SD for separated experiments and statistical analysis were made by paired Student's *t*-test or by one-way ANOVA for multiple factors analysis with SPSS 18.0 software. Differences were considered to be statistically significant when *P* < 0.05.

## 3. Results

### 3.1. Hydrogen Peroxide Exposure Affected the PE- and KCl-Induced Contraction

**Figure 1:**
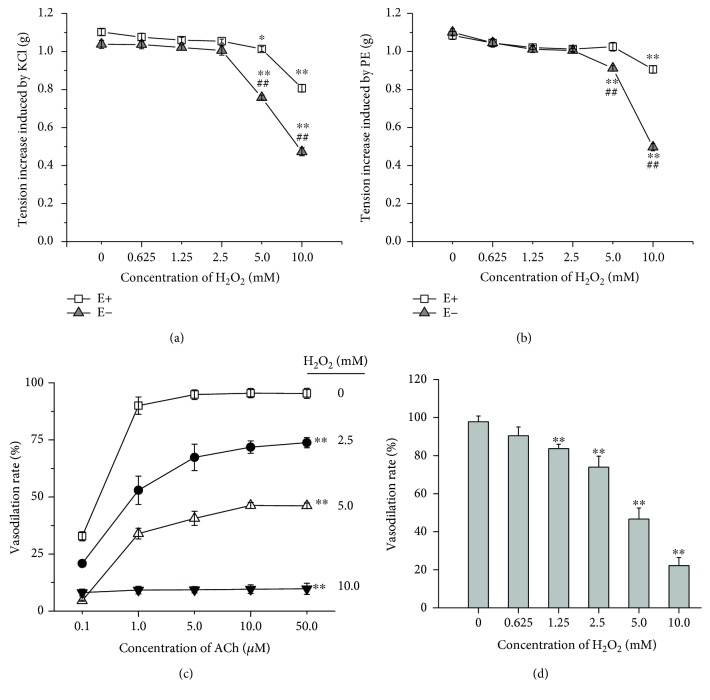
Effects of H_2_O_2_ exposure on PE- and KCl-induced contraction in rat aortic rings. (a) KCl induced contractile response in H_2_O_2_-treated aortic rings with (E+) or without (E−) endothelium. (b) PE induced contractile response in H_2_O_2_-treated aortic rings with (E+) or without (E−) endothelium. (c) ACh induced vasodilative response in H_2_O_2_-treated rat aorta with intact endothelium. (d) 5.0 mM H_2_O_2_ pulse treatment (10 min) induced the endothelium-dependent vasodilation impairments in rat aortic rings with intact endothelium. Data represents as means ± SD (*n* = 6). (a and b) ^∗^*P* < 0.05, ^∗∗^*P* < 0.01 versus the respective untreated group; ^##^*P* < 0.01 versus the endothelium-intact aortic rings treated with the same concentration of H_2_O_2_; (c and d) ^∗∗^*P* < 001 versus the untreated group.

The cumulative addition of H_2_O_2_ to 10.0 mM showed no obvious effect on the basal tension in endothelium-intact and endothelium-denuded aortic rings (data were not shown). The contraction response to PE or KCl was not affected till H_2_O_2_ reached 10.0 mM in endothelium-intact aortic rings. However, the 5.0 mM H_2_O_2_ pretreatment resulted in the significant decrease of the maximum contraction induced by PE or KCl in endothelium-denuded aortic rings (Figures 1(a) and 1(b)), which indicated that H_2_O_2_ induced more serious injury to the vascular smooth muscle in the endothelium-denuded aortic rings and the presence of the endothelium alleviated this injury.

A typical model regarding the concentration response curve of ACh-induced endothelium-dependent relaxation was impaired in H_2_O_2_-induced thoracic aorta compared with the control (Figures 1(c) and 1(d)) (control: pD_2_ = 7.00 ± 0.05, *E*_max_ = 90%; 2.5 mM H_2_O_2_: pD_2_ = 5.88 ± 0.12, *E*_max_ = 73%; 5 mM H_2_O_2_: pD_2_ = 4.52 ± 0.22, *E*_max_ = 47%).

### 3.2. Effect of RA on H_2_O_2_-Induced Endothelium-Dependent Vasodilation Impairments in Rat Aortic Rings

**Figure 2:**
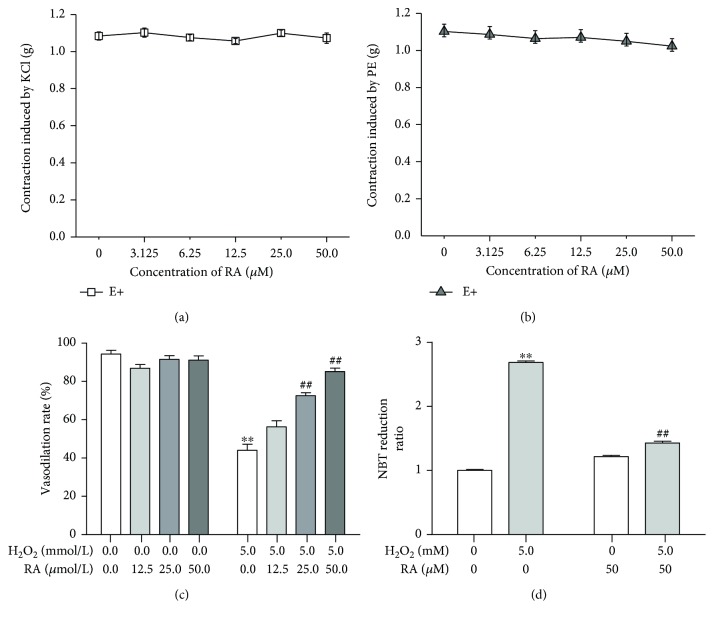
Rosmarinic acid alleviates the endothelial dysfunction induced by H_2_O_2_ in endothelium-intact rat aortic rings. (a) Rosmarinic acid (RA) pulse exposure showed no effect on the contraction induced by KCl in rat aortic rings. (b) Rosmarinic acid (RA) pulse exposure showed no effect on the contraction induced by PE in rat aortic rings. (c) Rosmarinic acid (RA) preincubation alleviated the endothelium-dependent vasodilation impairments induced by H_2_O_2_. (d) Rosmarinic acid (RA) cotreatment inhibited the NBT reduction induced by H_2_O_2_ in the endothelium-intact aortic rings. The results were expressed as the means ± SD (*n* = 6). ^∗∗^*P* < 0.01 versus the untreated control group; ^##^*P* < 001 versus the H_2_O_2_-treated group.

The cumulative concentration of RA to 50 *μ*M showed no effect on the contraction response to KCl or PE (Figures 2(a) and 2(b)), and the relaxation response to ACh (Figure 2(c)) in rat aortic rings as well, whereas it significantly alleviated the impairment of vasodilation reaction to ACh induced by H_2_O_2_ in a dose-dependent manner (Figure 2(c)) (*P* < 0.01). Because the oxidative stress mediates the endothelium injury and the NADPH oxidase is the main source of the endogenous reactive oxygen species, the O_2_^•−^ generation in the aortic rings was examined by the NBT reduction. The 5 mM H_2_O_2_ treatment significantly promoted the generation of the reduced NBT (formazan) in isolated rat aortic rings, while the 50 *μ*M RA almost entirely abolished the effect of H_2_O_2_ (Figure 2(d)).

### 3.3. eNOS Activation Was Involved in the Protection of RA against the Endothelial Dysfunction Induced by H_2_O_2_

**Figure 3:**
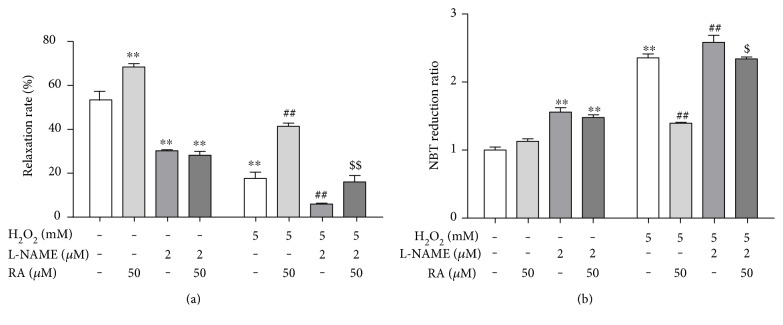
eNOS activation mediated the protection of rosmarinic acid on the endothelial dysfunction induced by H_2_O_2_ in rat aortic rings. The rat aortic rings were cotreated with eNOS inhibitor L-NAME (2.0 *μ*M) and RA (50 *μ*M) for 10 min, then exposed to H_2_O_2_ (5.0 mM) for another 10 min. The endothelial function was assessed by the endothelium-dependent vasodilation induced by acetylcholine (ACh, 10 *μ*M) (*n* = 6). (a) The relative endothelium-dependent vasodilation rate after exposure to RA, eNOS inhibitor L-NAME, and H_2_O_2_ in rat aortic rings. (b) The NBT reduction after exposure to RA, eNOS inhibitor L-NAME, and H_2_O_2_ in rat aortic rings. Data are presented as the means ± SD (*n* = 6). ^∗∗^*P* < 0.01 versus the untreated control group; ^##^*P* < 0.01 versus the H_2_O_2_-treated group; ^$^*P* < 0.05 and ^$$^*P* < 0.01 versus the H_2_O_2_- and RA-cotreated groups, respectively.

Given that NO is the most potent vasodilator and the modulator of intracellular oxidative status, and it is produced by the eNOS in endothelium [19], we explored the effect of eNOS activation in RA's protection against the endothelial dysfunction induced by H_2_O_2_. The pretreatment with the NOS inhibitor L-NAME alone significantly decreased the vasodilation induced by ACh (Figure 3(a)), while increased the NBT reduction (Figure 3(b)) in rat aortic rings. The L-NAME further increased the vasodilation impairment (Figure 3(a)), whereas it increased the NBT reduction (Figure 3(b)) in rat aortic rings induced by H_2_O_2_. Moreover, the L-NAME treatment abolished the RA's protection on the impairment of the endothelial-dependent relaxation injured by H_2_O_2_ (Figure 3(a)). And the decrease of NBT reduction induced by RA was also reversed by L-NAME in H_2_O_2_-treated aortic rings (Figure 3(b)).

### 3.4. AMPK Activation Was Involved in the Protection of RA against the Endothelial Dysfunction Induced by H_2_O_2_

**Figure 4:**
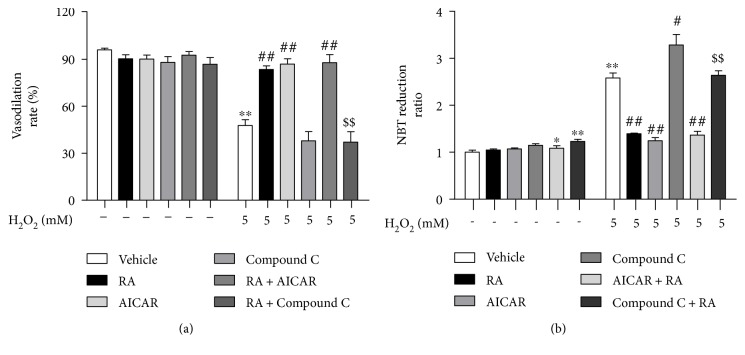
AMPK activation mediated the protection of rosmarinic acid against the endothelial dysfunction induced by H_2_O_2_ in rat aortic rings. The rat aortic rings were cotreated with AMPK activator AICAR (50 *μ*M, 10 min) or inhibitor compound C (10 *μ*M) and RA (50 *μ*M) for 10 min, then exposed to H_2_O_2_ (5.0 mM) for another 10 min. The endothelial function was assessed by the endothelium-dependent vasodilation induced by acetylcholine (ACh, 10 *μ*M). (a) The relative endothelium-dependent vasodilative rate after exposure to RA, AMPK modulator, and H_2_O_2_ in rat aortic rings. (b) The NBT reduction after exposure to RA, AMPK modulator, and H_2_O_2_ in rat aortic rings. Data are presented as the means ± SD (*n* = 6). ^∗^*P* < 0.05 versus the untreated control group; ^∗∗^*P* < 0.01 versus the untreated control group; ^##^*P* < 0.01 versus the H_2_O_2_-treated group; ^$$^*P* < 0.01 versus the H_2_O_2_- and RA-cotreated groups.

AMPK is a crucial cellular energy sensor which senses change in the intracellular AMP/ATP ratio. It is also an intracellular stress sensor that is regulated by oxidative stress and other stresses that result in diminished cellular ATP levels. AMPK is one of the key modulators of eNOS in the endothelium and involved in the endothelial dysfunction induced by oxidative stress resulted from NADPH oxidase upregulation [20]. Here, we investigated the roles of AMPK in RA's protection to endothelial dysfunction induced by H_2_O_2_. As shown in Figure 4(a), compared with control, the response to ACh was similar to the AICAR- and RA-treated groups (*P* < 0.01). Similarly, activation of AMPK by AICAR decreased the NBT reduction. Whereas, the beneficial effect of RA on endothelium-dependent vasodilatation in rats was partly attenuated in the presence of compound C, a well-characterized AMPK inhibitor, reduced AMPK activity, and enhanced NBT reduction at 10 *μ*M (Figure 4(b)). Furthermore, when compound C was combined with H_2_O_2_, it intensified the NBT reduction. Mechanistically, we found that AMPK activated and increased the protection of RA on endothelial dysfunction (Figure 4(b)).

### 3.5. RA Treatment Improved Endothelial Dysfunction in HAEC via AMPK/eNOS Pathway

**Figure 5:**
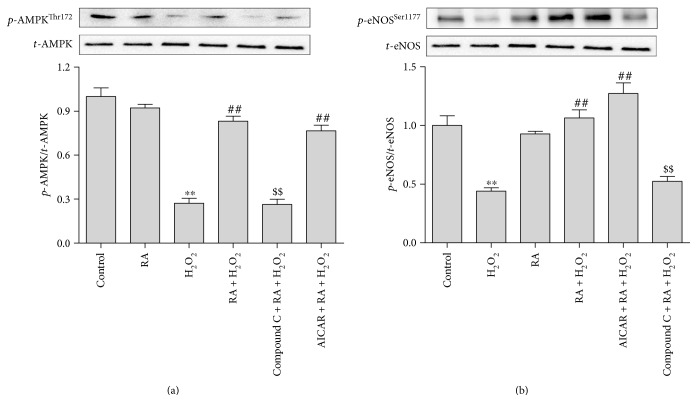
Rosmarinic acid induced the phosphorylation of AMPK and eNOS in HAEC cells. The HAEC cells were pretreated with the AMPK inhibitor compound C (10 *μ*M) or activator AICAR (50 *μ*M) combined with RA (50 *μ*M) for 10 min prior to another 10 min exposure with H_2_O_2_ (5 mM). The total AMPK (*t*-AMPK), phosphorylated AMPK at Thr172 site (*p*-AMPK^Thr172^), and the total eNOS (*t*-eNOS) and the phosphorylated eNOS at the Ser1177 site (*p*-eNOS^Ser1177^) were determined by Western blot. The results is quantified as the relative ratio of the phosphorylated protein/total protein. The values are presented as the means ± SD; *n* = 3. ^∗∗^*P* < 0.01 versus the untreated control group; ^##^*P* < 0.01 versus the H_2_O_2_-treated group; ^$$^*P* < 0.01 versus the H_2_O_2_- and RA-cotreated groups.

In order to further ascertain the relationship of AMPK and eNOS in the RA's effect, the AMPK-eNOS signal pathway activation was investigated by their phosphorylation in human aortic endothelial cells (HAEC) *in vitro*. As shown in Figure 5, the expression levels of total AMPK and eNOS remained unchanged. And the 50 *μ*M RA single treatment had no obvious effect on the AMPK and eNOS phosphorylation, while the 5 mM H_2_O_2_ treatment significantly downregulated the phosphorylation of AMPK and eNOS at Thr172 and Ser1177, respectively (*P* < 0.01), in HAEC cells. The cotreatment with RA significantly reversed the decrease of AMPK and eNOS phosphorylation induced by H_2_O_2_. The AMPK's inhibitor, compound C cotreatment, abolished the RA's upregulation of AMPK and eNOS phosphorylation in H_2_O_2_-treated HAEC cells. However, the AMPK agonist showed no more synergistic effect with RA. The results suggest that the AMPK phosphorylation played key roles in the protection effects of RA on H_2_O_2_-induced injury in HAEC.

## 4. Discussion

Endothelial dysfunction resulted from oxidative stress is the key initiating factor in almost all vascular events. Niethammer et al. found that the extracellular H_2_O_2_ generated by dual oxidase (Duox) reached 50 *μ*M after 20 min of wounding near the wound margin in zebrafish larvae, which constructed a concentration gradient and mediated the rapid recruitment of leukocytes to the wound [7]. Although the oxidative burst derived from the neutrophil activation in the inflammation will produce more ROS, there is no accurate concentration data of H_2_O_2_ reported. Here, we investigated the dose-effect relationship of H_2_O_2_ on the function of endothelium and vascular smooth muscle. In order to exclude the direct reaction of H_2_O_2_ with PE or ACh, the aortic rings were incubated in fresh K-H solution for 10 min and redepolarized with KCl after H_2_O_2_ pulse treatment for 10 min. The results showed that the vascular smooth muscle reactivity to PE or KCl in endothelium-denuded aortic rings were more vulnerable to H_2_O_2_ (at 5.0 and 10.0 mM) than in the endothelium-intact aortic rings (Figure 1()), which indicated that the presence of endothelium protected the vascular smooth muscle from the oxidative injury induced by high concentration of H_2_O_2_. Moreover, the 2.5 mM H_2_O_2_ pretreatment resulted in the significant decrease of the vasodilative reaction to ACh, which demonstrated that the endothelial cells were more sensitive to H_2_O_2_ than the vascular smooth muscle cells.

The previous work of Sotnikova et al. proved that RA significantly improved the endothelium-dependent vasodilation in diabetic rat aorta, which might be mediated by its antioxidative and anti-inflammation properties [8]. The present study proves that RA improves the impairments of endothelial-dependent vasodilation caused by H_2_O_2_ in normal rat aorta (Figure 2()). NADPH oxidase is the major source of reactive oxygen species in endothelial cells and vascular smooth cells [21]. Besides, it has been proved that endothelial-dependent relaxation was effectively improved after the deletion of Nox2, which implicates that the endothelial dysfunction might be associated with Nox2 overexpression [22]. H_2_O_2_-induced endothelial-dependent relaxation impairment is associated with the increased production of superoxide anion (O_2_^•−^). It has been proved that H_2_O_2_ could activate NADPH oxidase in a dose- and time-dependent manner in respiration rate [23], and the oxidative injury to endothelium resulted from the excess ROS is the key mediator of endothelial impairment in atherosclerosis and diabetes [24]. Our work also proved that the pulse treatment with H_2_O_2_ significantly increased the NBT reduction in rat aorta and RA cotreatment significantly reversed the effects of H_2_O_2_, which demonstrates that the antioxidative activity is involved in the RA's protective effects on the endothelial function.

The endothelium-dependent vasodilation impairment is believed to be the consequence of a decreased bioavailability of nitric oxide (NO), an important endothelium-derived relaxing factor. The superoxide derived from NADPH oxidase could rapidly react with NO to form the stable peroxynitrite anion (ONOO^−^), which will result in the decline of NO bioavailability. The other reason for NO signal dysfunction might lie in the eNOS expression and activation impairments. In our experiments, L-NAME partially decreased the phosphorylation of eNOS and antagonized the protective effects of RA on the endothelium dysfunction induced by H_2_O_2_, while it exacerbated the ROS formation in H_2_O_2_-treated rat aorta. These results revealed that the effects of RA might be associated with the NO synthesis.

In the recent years, AMPK is demonstrated to improve vascular function by activating eNOS [25]. In addition to regulating energy metabolism, AMPK exerts anti-inflammatory and antioxidative activities [26, 27]. Previous studies indicated that AMPK activation improved the endothelial function [28]. Here, we found that the AMPK agonist AICAR single treatment possessed the similar protective effects against endothelial dysfunction and oxidative stress induced by H_2_O_2_ in rat aorta as well as RA did, while the combination of RA and AICAR showed no further beneficial effect. The cotreatment with AMPK inhibitor compound C abolished the effects of RA, which further proved that the AMPK activation played a key role in the RA's effects. Hu et al.'s work revealed that H_2_O_2_ induced the bidirectional modulation in eNOS through the Akt and AMPK in a time- and dose-dependent way [29]. We also found that the Akt inhibitor LY294002 cotreatment significantly abrogates the RA's protection from the endothelium-dependent vasodilation impairments (Figure 1S available online at https://doi.org/10.1155/2017/7091904) induced by H_2_O_2_. And the RA significantly restored the phosphorylation downregulation at the Ser473 site of the Akt protein (Figure 2S). It indicated that the Akt signal pathway was involved in the RA's protective effects, while the interaction of AMPK and Akt in RA's effects needed to be investigated further.

In summary, this study demonstrated that RA significantly improved H_2_O_2_-induced endothelial dysfunction and the activation of AMPK-eNOS pathway was involved in the RA's effects. However, whether the modulating effects of RA is dependent on its direct activation of AMPK-eNOS pathway or its modulation on the oxidative status still remains unclarified in the present work. It needs further investigations to identify the underlying mechanisms of RA's protection on the endothelial function.

## Supplementary Material

Figure 1S. The influence of Akt level in the protection of RA against H_2_O_2_-induced endothelial dysfunction in aorta rings. Figure 2S. The influence of Akt level in the protection of RA against H_2_O_2_-induced endothelial dysfunction in HAEC.

## Abbreviations

RA: Rosmarinic acid
K-H: Krebs-Henseleit
AICAR: 5-Aminoimidazole-4-carboxamide-1-*β*-D-ribofuranoside
PE: Phenylephrine
ACh: Acetylcholine
EC+: Endothelium-intact aortic rings
EC−: Endothelium-denuded aortic rings
L-NAME: NG-nitro-L-arginine methyl ester
eNOS: Endothelial nitric oxide synthase
I/R: Ischemia and reperfusion
HAEC: Human aortic endothelial cells.
