# Supplementary material for: Rosmarinic Acid Alleviates the Endothelial Dysfunction Induced by Hydrogen Peroxide in Rat Aortic Rings via Activation of AMPK
Source: Oxid Med Cell Longev. 2017 Aug 13;2017:7091904. doi: 10.1155/2017/7091904 (PMC5572610; doi:10.1155/2017/7091904)
Supplement: Supplementary file 1 — Figure 1S. The influence of Akt level in the protection of RA against H2O2-induced endothelial dysfunction in aorta rings. Figure 2S. The influence of Akt level in the protection of RA against H2O2-induced endothelial dysfunction in HAEC. [file 7091904.f1.pdf]

Supplementary data

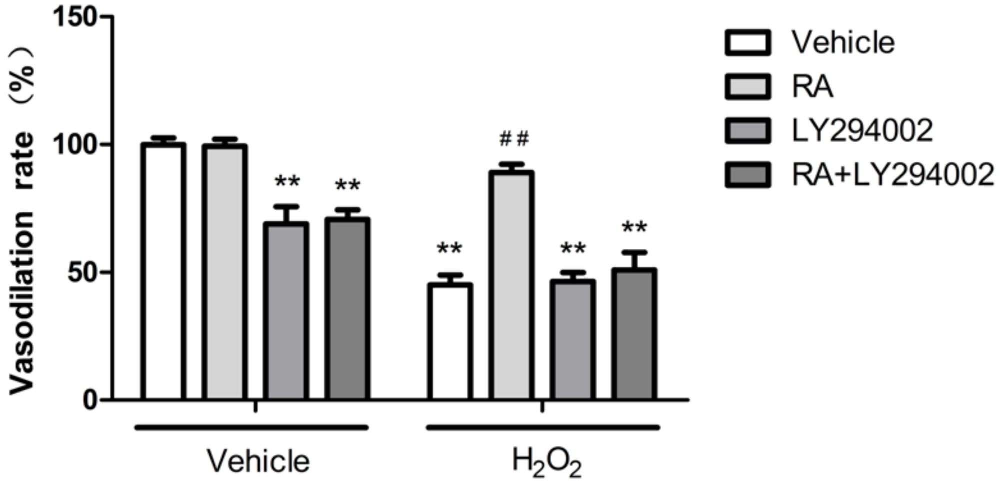

Fig 1S. The influence of Akt level in the protection of RA against H2O2-induced endothelial dysfunction in aorta rings. The rat aorta was pretreated withLY294002, RA for 10 min, added H_2O_2 for another 10 min. RA (50 μM, 10 min) has improved endothelial dysfunction in H_2O_2-induced rats, but the effects were partly suppressed by Akt inhibitor (LY294002, 25 μM), The vasodilation was assessed by exposure acetylcholine (ACh) (n=6). ** P<0.01 vs control group.[##] P<0.01 vs H_2O_2 group.

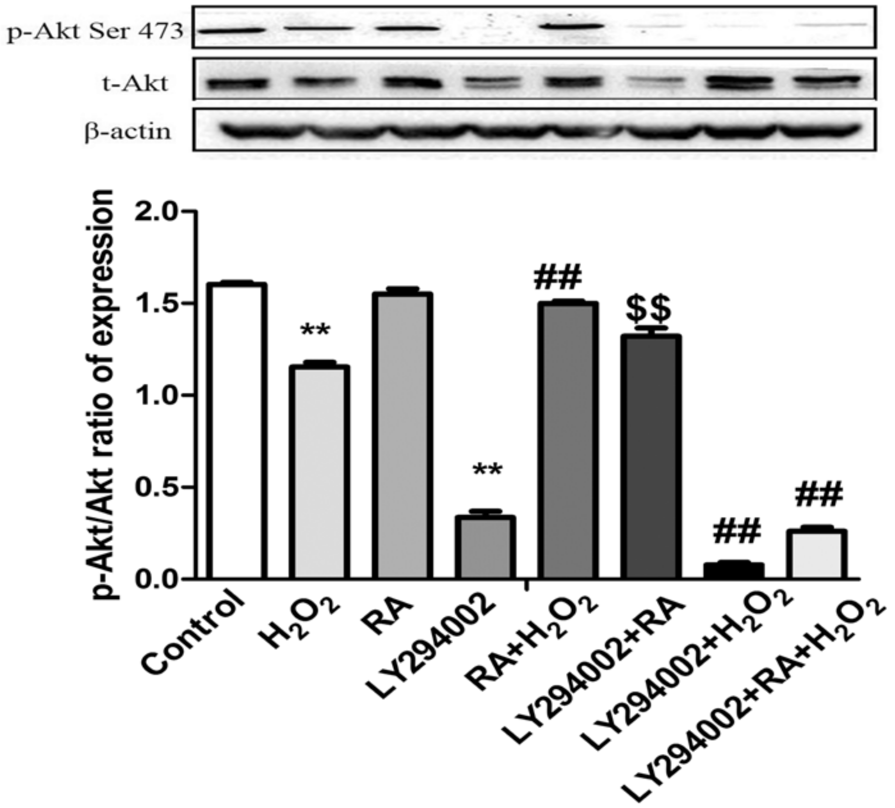

Fig. 2S The influence of Akt level in the protection of RA against H_2O_2-induced endothelial dysfunction in HAEC. t the HAEC were pretreated with the Akt inhibitor Ly294002(25 μM) 10 min prior to incubation for an additional 10 min with RA (50 μM) and H_2O_2 (5 mM). Phosphor-Akt, Akt levels were determined by western blot. The quantifications of the related phosphor-Akt, Akt are shown. The values are presented as the means ± SD, ** *P*<0.01 vs control group, $^{\#\#}$ *P*<0.01 vs H_2O_2 group.$$ *P*<0.01 vs RA group.

Materials and Methods

Chemicals

Rosmarinic acid, acetlcholin (ACh), Compound C，5-Aminoimidazole-4-carboxamide-1-β-D-ribofuranoside (AICAR), LY294002 were purchased from Sigma Chemical Co (St. Louis, MO, USA); phenylephrine (PE) and *l*-N-nitro arginine methyl ester (L-NAME) was purchased from Aladdin Industrial Co (Shanghai, China). The other reagents were of analytical purity. Akt inhibitor LY294002 was dissolved in dimethyl sulfoxide (DMSO) Polyclonal antibodies against AMPK, phosphor-AMPK(Thr172) Akt, phospho-Akt (Ser473), were obtained from Cell Signaling Technology (Beverly, Mass), eNOS, phospho-eNOS (Ser1177) were purchased from Abcam.

Endothelial dysfunction induced by H_2O_2 and the RA treatment in rat aortic rings

After 10 min equilibration with new K-H solution, the aortic rings were pretreated with various concentrations of H_2O_2 (2.5, 5.0 and 10.0 mM) for 10 min. Following washout of H_2O_2 the aortic rings was depolarized with 80 mM KCl for 2 times. After returning to baseline tension, rings were allowed to equilibrate for 20 min and then the contraction were induced with PE (1μM) till a stable plateau in tension. Then each ring was exposed to increasing concentration of ACh (10^{-3}, 10^{-2}, 10^{-1}, 1, 5, 10, 50 μM) to generate does-dependent relaxation response. In the RA intervention effect investigation, the aortic rings were incubated with various concentrations (50.0 μM, 25 μM, 12.5 μM) of RA 10 min prior to exposure to 5 mM H_2O_2. Thereafter, a second vasodilation reactivity to ACh was obtained to evaluate the integrity of the endothelium after PE-induced contration. In order to investigate the roles Akt and AMPK in H_2O_2 induced endothelium dysfunction, the aortic rings were separately pretreated for 10 min with two inhibitors of Compound C and LY294002 before the exposure to H_2O_2 (5 mM).

Cell culture and treatment

HAEC (Human aortic endothelial cells) were purchased from Thermo Fisher Scientific Inc. and cultured in Dulbecco’s Modified Eagle Medium (GIBCO) supplemented with L-glutamine, pyridoxine hydrochloride, 110 mg/L sodium pyruvate and penicillin. Streptomycin and amphotericin at 37 ℃ in a humidified atmosphere of 5% CO_2. Cells cultured up to six or fewer passages were first grown to confluence before exposure to H_2O_2 (5 mM) for 10 min, and stimulated by RA (50 μM) containing H_2O_2 (5 mM) for 10 min, to clarify the activity of AMPK on the

expression of the phosphor-eNOS. Therefore, cells were treated with Compound C (inhibitor of AMPK) with H_2O_2, and in the presence of RA for 10 min. Next, in order to see if rosmarinic acid-mediated Akt activation was responsible for the enhancement of eNOS phosphorylation, cells were treated with LY294002 (Akt inhibitor) in the presence of rosmarinic acid found that LY294002 completely abrogated rosmarinic acid-mediated increase in Akt phosphorylation.

Western blotting analysis

After lysis of the cells, the protein samples (25 μg/lane) were resolved by electrophoresis on 10% sodium dodecyl sulfate (SDS)-polyacrylamide gels and then transferred to nitrocellulose membranes. The membranes were inbubated in blocking buffer and then incubated with more of the following primary antibodies. Anti-AMPK (1∶1000, Cell signaling, MA, USA), anti-phospho-AMPK (Thr 172) (1∶1000, Cell signaling), anti-phospho-Akt(ser 473)(1:1000, Cell signaling), anti-Akt(1:1000, Cell signaling), anti-phospho endothelial nitric oxide synthase (eNOS, ser 1177), anti-endothelial nitric oxide synthase (1∶1000, Cell signaling). Thereafter, the membranes were washed and incubated with horseradish peroxidase-conjugated secondary antibodies. (1：2000, Cell signaling).

Results

Akt level was involved in the protection of Rosmarinic acid in aorta rings.

As presented in Fig. 6, H_2O_2-induced aorta relaxation responses were obviously attenuated in rings compared with control. Treatment with Rosmarinic acid (50 μM) significantly improved H_2O_2-induced vasoconstriction in aortic rings. The nicer effect of Rosmarinic acid (50 μM) on vasodilation response to H_2O_2 was abolished by administration with LY294002 (25 μM).

Akt level was participated in the protection of RA in HAEC.

Fig. 7 describes that phosphorylations of Ser473-Akt and Akt were greatly decreased in H_20_2-treated HAEC compared with controls, but significantly elevated in RA-treated from HAEC cells. In contrast, after pretreatment with RA (50μM) phosphorylations of Ser473－Akt and Akt levels were unchanged compared with controls, respectively. In addition, the phosphoinositide 3-kinase (PI 3-K)/Akt intracellular signalling pathway inhibitor LY-294002 inhibited the effect of RA on Akt phosphorylation induced by H_2O_2. Suggesting that RA accelerated the serine phosphorylations of Akt and eNOS inH_2O_2-induced damage in HAEC, which was abolished by Akt inhibitor(LY294002).

Discussion

As shown in our study, treatment with RA strikingly attenuated H_2O_2-induced damage of aortic vascular tone in mice. This favorable effect of RA was abolished by PI3K/Akt inhibitor LY294002, indicating that RA revealed a positive-regulatory role against the actions of H_2O_2 involving the PI3K/Akt-eNOS signaling pathway. These findings have given strong support to the concept that the beneficial effects of RA on aortic vascular tone are likely due to activation of phosphorylation of Ser473-Akt in mice. In contrast, in endothelial cells from LY294002 treatment, H_2O_2 induced activation of Akt phosphorylation was abolished, supporting the role of Akt in RA-mediated aortic vascular tone and endothelial cells protection. Thus, diminished expression of Akt observed in the current study may be, at least in part, responsible for reduced phosphorylations of Ser473-Akt in the HAEC. Generalising from the results, RA may act as a “good Regulatory”in the vasculature, serving to modulate the abnormal aortic vascular tone in response toH_2O_2 and acetylcholine by enhancing the phosphorylations of Ser473-Akt, suggesting that RA may be an important regulator of vascular function and exert a protective role in H2O2-induced endothelial cells impaired.
